# Inhalable Anti-EGFR Antibody-Conjugated Osimertinib Liposomes for Non-Small Cell Lung Cancer

**DOI:** 10.3390/pharmaceutics16111444

**Published:** 2024-11-12

**Authors:** Apoorva Daram, Shruti S. Sawant, Dhwani A. Mehta, Carlos A. Sanhueza, Nitesh K. Kunda

**Affiliations:** Department of Pharmaceutical Sciences, College of Pharmacy and Health Sciences, St. John’s University, New York City, NY 11439, USA

**Keywords:** non-small cell lung cancer, osimertinib, cetuximab, immunoliposomes, inhalation therapy

## Abstract

**Background**: Non-small cell lung cancer (NSCLC) is a leading cause of cancer deaths globally. The most extensive treatment is Tyrosine Kinase Inhibitors (TKIs) that target epidermal growth factor receptor (EGFR) overexpression. Osimertinib, a third-generation TKI is approved to target EGFR exon 19 deletions or exon 21 L858R mutations. However, resistance is inevitable due to emergence of triple mutations (sensitizing mutations, T790M and C797S). To overcome this challenge, a combinatorial approach was used wherein Osimertinib liposomes were conjugated with cetuximab (CTX), an anti-EGFR monoclonal antibody, to improve drug efficacy and delivery. Additionally, pulmonary administration was employed to minimize systemic toxicity and achieve high lung concentrations. **Methods**: Osimertinib liposomes (OB-LPs) were prepared using thin film hydration method and immunoliposomes (CTX-OB-LPs) were prepared by conjugating the OB-LPs surface with CTX. Liposomes were characterized for particle size, zeta-potential, drug loading, antibody conjugation efficiency, in vitro drug release, and aerosolization performance. Further, the in vitro efficacy of immunoliposomes was evaluated in H1975 cell line. **Results**: Immunoliposomes exhibited a particle size of 150 nm, high antibody conjugation efficiency (87%), efficient drug release, and excellent aerosolization properties with an aerodynamic diameter of 3 μm and fine particle fraction of 88%. Furthermore, in vitro studies in H1975 cells showed enhanced cytotoxicity with CTX-OB-LPs displaying 1.7-fold reduction and 1.2-fold reduction in IC_50_ compared to Osimertinib and OB-LPs, respectively. The CTX-OB-LPs also significantly reduced tumor cell migration and colonization compared to Osimertinib and OB-LPs. **Conclusions**: These successful results for EGFR-targeting inhalable immunoliposomes exhibited potential for contributing to greater anti-tumor efficacy for the treatment of non-small cell lung cancer.

## 1. Introduction

Lung cancer is the second most common cancer and is the leading cause of cancer mortality [1]. Histologically, lung cancer is divided into small cell (SCLC) and non-small cell lung cancer (NSCLC), with NSCLC accounting for approximately 85% of the cases [2]. However, despite advances in treatment options such as immunotherapy and targeted therapy, the overall survival rate is merely 20–25%, and even patients diagnosed at an early stage (stage I and II) have an overall survival rate of ~40% [3,4,5].

Advances in molecular profiling have led to the discovery of specific mutations that occur in lung cancer, and targeted therapies against these mutations have shown tremendous success for NSCLC treatment [6]. Among the earliest identified and targeted mutations were those found in the epidermal growth factor receptor (EGFR) gene, which is overexpressed in NSCLC [7]. EGFR is a transmembrane receptor with an extracellular domain, transmembrane region, and intracellular tyrosine kinase domain [8]. EGFR becomes activated upon ligand binding, dimerization, and phosphorylation. This activation triggers cellular signaling pathways resulting in cell proliferation, survival, and migration, meaning uncontrollable multiplication of cells [7,9]. The EGFR receptor has been involved in the progression of various tumors and, therefore, has been a promising target for non-small cell lung cancer [10]. EGFR can be blocked either by using small molecule tyrosine kinase inhibitors (TKIs) like gefitinib, afatinib, dacomitinib, and osimertinib that bind to the intracellular domain of the receptor or by using monoclonal antibodies (mAbs) such as cetuximab that bind to the extracellular domain of the receptor [11].

Osimertinib (OB) is an approved TKI-based therapy for patients with EGFR exon 19 deletions or exon 21 L858R mutations. In addition, monoclonal antibodies like cetuximab that bind to the extracellular domain of the EGFR are used as an immunotherapy option. In the current literature, there are different approaches to improve the efficacy of TKIs [12,13]. An effective strategy could be to use a combination of TKI and cetuximab [14]. Therefore, our work highlights the development of a liposomal drug delivery system using a combination of osimertinib and an antibody to improve overall efficacy. A clinical study has reported that a combination therapy of osimertinib and cetuximab for a patient with an EGFR exon 20 mutation demonstrated efficient anti-tumor efficacy and that this promising result highlights the potential of this combination therapy [15].

A nanoparticulate delivery system is essential to aid the delivery of two drugs, a small molecule and an antibody, to the target site. The main challenges associated with conventional therapies include a lack of tumor specificity which further results in serious toxicities. This non-specific action and poor distribution results in inadequate drug concentrations at the tumor site. To overcome the abovementioned challenges, nanoparticulate systems are conjugated with antibodies to target the tumor site, thereby increasing the intracellular concentration in cancer cells and offsetting toxicity in healthy cells [16,17]. In our study, we utilize a liposomal delivery system owing to its numerous advantages such as biocompatibility, controlled release, targeted delivery, protection of the drug against rapid degradation, and improved pharmacokinetics, among others [18,19]. Additionally, the unique structure and modifiable surface of the liposomes enable the efficient and safe delivery of the drugs across various applications [20].

Regardless of the benefits of using liposomal delivery systems, oral and intravenous administration of these systems often results in a poor biodistribution to the lungs. However, direct administration to the target site, i.e., the lungs, would be beneficial to achieve higher drug concentrations at the primary tumor site. Furthermore, inhalation therapies are non-invasive, require lower drug doses, bypass the first pass effect, and are associated with a higher degree of patient compliance compared to oral and injectable drug delivery systems [21].

Resistance to EGFR-TKI therapy is inevitable and osimertinib is an approved NSCLC therapy for patients bearing the EGFR exon 19 deletions or exon 21 L858R mutations. However, the development of T790M mutations on the EGFR domain can cause resistance to osimertinib. To help overcome OB resistance, the main objective of this research is the development of anti-EGFR antibody-conjugated OB liposomes, which serve the dual purpose of acting as a targeting ligand and providing synergistic anti-cancer efficacy.

## 2. Materials and Methods

### 2.1. Materials and Cell Line

Osimertinib (OB) was purchased from MedChemExpress (Monmouth Junction, NJ, USA). The lipids 1,2-dipalmitoyl-sn-glycero-3-phosphocholine (DPPC) and cholesterol were procured from Avanti Polar Lipids (Alabaster, AL, USA). The lipid cholesterol-PEG-COOH (cholesterol functionalized polyethylene glycol with a terminal carboxylic group) was purchased from Nanosoft Polymers (Winston-Salem, NC, USA). Acetonitrile (HPLC grade), methanol (HPLC grade), orthophosphoric acid (HPLC grade), and water (HPLC grade) were purchased from Fisher Scientific (Hampton, NH, USA). Phosphate buffer saline (PBS) was acquired from Corning Inc. (New York, NY, USA). The chemical reagents, N-hydroxysulfosuccinimide sodium salt (NHS) and 3-(3-dimethylaminopropyl)-1-ethyl-carbodiimide hydrochloride (EDC) were purchased from Chem-Impex International Inc. (Wood Dale, IL, USA). Both 3-(4,5-dimethylthiazol-2-yl)-2,5-diphenyltetrazolium bromide (MTT) and crystal violet were obtained from Fisher Scientific (Hampton, NH, USA). Cetuximab was purchased from BioXCell (Lebanon, NH, USA).

The human non-small cell lung cancer adenocarcinoma cell line, NCI-H1975 [H1975] (ATCC CRL-5908), was procured from the American Type Culture Collection (Manassas, VA, USA). The H1975 cells were grown in Roswell Park Memorial Institute (RPMI) media (Corning Inc., New York, NY, USA) supplemented with 20% fetal bovine serum (Atlanta Biologicals, Flowery Branch, GA, USA), 1% penicillin–streptomycin (Corning Inc., New York, NY, USA), and incubated at 37 °C under 5% carbon dioxide.

### 2.2. Osimertinib Liposomes/Unconjugated OB Liposomes (OB-LPs)

**Figure 1:**
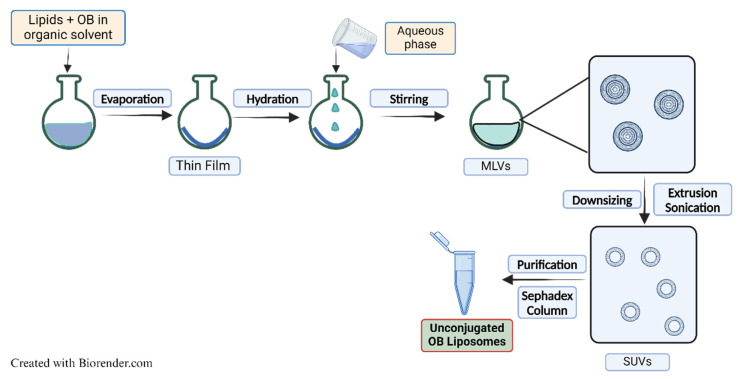
Schematic diagram for liposomal formulation preparation.

Osimertinib liposomal formulation (OB-LPs) was prepared using a thin film hydration method with slight variations [22]. Osimertinib (OB) was incorporated into liposomes by passive loading. The lipids, DPPC, cholesterol, and cholesterol-PEG-COOH, with a total lipid concentration of 23 mM, were used in a molar ratio of 7.6:2.3:0.1, respectively. The lipids (30 mg) and drug (0.5 mg/mL) were dissolved in 5 mL methanol and transferred to a round bottomed flask and connected to a rotary evaporator (R-100 Rotavapor, Buchi, New Castle, DE, USA) for solvent evaporation at 45 °C to obtain a dried thin film. The obtained thin film was hydrated with PBS to obtain multilamellar vesicles. The multilamellar vesicles were reduced to single unilamellar vesicles via probe sonication (Q500 Sonicator, QSonica Sonicators, Newtown, CT, USA) for 4 min, on an ice bath, at 40% amplitude pulsed sonication. The formulation was passed through a size exclusion Sephadex G-25 PD-10 column (Cytiva, Marlborough, MA, USA) with PBS as the eluent to separate the unencapsulated drug. The collected liposomal formulation was then extruded through a 0.2 μm polycarbonate membrane using a mini extruder (Avanti Polar Lipids, Alabaster, AL, USA) to obtain OB-LPs (Figure 1). Blank liposomes (blank LPs) were similarly prepared, but without OB.

### 2.3. Targeted Osimertinib Liposomes/Immunoliposomes (with Cetuximab) (CTX-OB-LPs)

The targeted osimertinib liposomal formulation (CTX-OB-LPs) with cetuximab was prepared using the method mentioned in Section 2.2, with additional steps. Following extrusion, the activating agents, sulfo-N-hydroxy succinimide (NHS) and ethyldiicarbodimide (EDC) in a molar ratio of 2.5:1, respectively, were added to the liposomal suspension and mixed for 15 min on a rotating mixer. The suspension was, again, passed through a size exclusion Sephadex G-25 column equilibrated with PBS to remove any byproducts formed during the EDC-NHS reaction. Subsequently, the antibody solution (1 mg) was mixed with the liposomal suspension (with the activated carboxylic groups) and incubated for 4 h at 25 °C using a rotating mixer. After, the unbound antibody was separated by loading the liposomes into a Spectra Por Float-A-Lyzer G2 (300 kDa MWCO, Spectrum Laboratories Inc., Rancho Dominguez, CA, USA) dialysis device, immersed in 100 mL of PBS, and maintained under continuous stirring for 2 h. The final conjugated liposomes were collected and analyzed to determine the antibody conjugated onto the liposomal surface. Empty CTX-LPs (i.e., CTX-conjugated liposomes) were similarly prepared without OB.

### 2.4. Liposome Characterization (OB-LPs and CTX-OB-LPs)

#### 2.4.1. Particle Size Distribution and Zeta Potential

The particle size, polydispersity index, and zeta potential of the liposomes were determined through dynamic light scattering (DLS) using the Malvern Zetasizer (Malvern Panalytical, Malvern, UK), as published by us previously [22]. Briefly, the DLS measurements were recorded at room temperature by diluting 20 µL of the liposomal suspension with 1500 µL of deionized water (*n* = 3).

#### 2.4.2. Encapsulation Efficiency and Drug Loading

The encapsulation efficiency and drug loading of OB were calculated using high performance liquid chromatography (HPLC), as previously published by us [22].

#### 2.4.3. Antibody Conjugation Efficiency 

Antibody conjugation efficiency refers to the number of antibodies that attach to the liposomal surface relative to the initial amount added to the liposomal suspension. This was determined using the micro Bicinchoninic acid (BCA) protein assay kit as per the manufacturer’s protocol (ThermoFisher Scientific, Waltham, MA, USA). Briefly, the conjugated liposomes were incubated for 2 h at 37 °C with the working reagents, and the absorbances were analyzed at 570 nm using a microplate reader (Synergy H1, BioTek, Winooski, VT, USA).

#### 2.4.4. In Vitro Drug Release

In vitro drug release studies for OB were performed using the dialysis method in media containing PBS at a pH of 7.4 with 1% Tween 80^®^. Briefly, 0.5 mL of the liposomal formulation was loaded into a dialysis cassette (7000 MWCO, ThermoFisher Scientific, Waltham, MA, USA) and immersed in 120 mL of release media maintained at 37 °C. Samples were collected at predetermined time intervals (1 h, 2 h, 4 h, 6 h, 8 h, 10 h, 24 h, 30 h, and 48 h) from the release media and replaced with fresh media (2 mL) to maintain sink conditions. The amount of OB released into the media at different time points was analyzed using the HPLC method previously published by us [22].

#### 2.4.5. Powder X-Ray Diffraction (PXRD)

The OB-LPs and CTX-OB-LPs were freeze-dried overnight and the encapsulation of OB within the liposomes was further confirmed via Powder X-ray diffraction analysis. Briefly, PXRD studies were conducted using XRD6000 (Shimadzu, Kyoto, Japan). The sample was uniformly dispersed onto a micro-sample glass holder and analyzed at a scan range of 10°–80°, with a scanning speed of 2° (2θ/min).

### 2.5. In Vitro Aerosol Performance

The pulmonary deposition of CTX-OB-LPs was evaluated using the next generation impactor (NGI Model 170, MSP Corporation, Shoreview, MN, USA). The NGI plates and the adaptor were refrigerated at 4 °C for approximately 90 min prior to usage in the analysis. This was done to ensure minimal evaporation of the nebulized sample. Two mL of the liposomal formulation was loaded into a PARI LC PLUS^®^ nebulizer cup for nebulization using a PARI FAST-NEB compressor system. The airflow rate was maintained at 15 L/min during the sample run using a HCP5 vacuum pump (Copley Scientific, Nottingham, UK). The equipment was primed for 30 s and then nebulized for 4 min. Post-run samples were collected from each stage from 1 through 8, including the throat piece and mouthpiece, using a 1:1 ratio of ACN:water solvent mixture. The collected samples were centrifuged, and the supernatant was analyzed using HPLC. Various parameters such as mass median aerodynamic diameter (MMAD), fine particle fraction (FPF), and geometric standard deviation (GSD) were calculated to determine the aerosolization performance.

#### Effect of Nebulization on Liposomal Integrity

The stability of the liposomes after nebulization was determined by assessing the physicochemical characteristics of the immunoliposome formulation (CTX-OB-LPs), pre- and post-nebulization. Briefly, the liposomal formulation was nebulized using the PARI LC PLUS^®^ nebulizer and the aerosolized formulation was collected into a beaker. The particle size and zeta potential of the formulation, pre- and post-nebulization, were analyzed using the abovementioned method in Section 2.4.1.

### 2.6. Antibody–Antigen Binding Kinetics—LSPR (CTX and CTX-OB-LPs)

The binding affinity of immunoliposomes was determined by localized surface plasmon resonance (LSPR) using an OpenSPR Rev4 instrument (Nicoya Lifesciences, Kitchener, ON, Canada) at 25 °C in PBS buffer, at a pH of 7.4 containing 1% BSA and 0.05% Tween 20^®^. The epidermal growth factor receptor protein (EGFR, 50 μg/mL) was immobilized on a CM5 high-capacity carboxyl sensor chip (Nicoya Lifesciences, Kitchener, ON, Canada) using standard carbodiimide coupling, where the following solutions were sequentially injected: 0.4 M EDC and 0.1 M NHS to activate the carboxyl groups on the sensor chip surface, followed by 50 μg/mL EGFR-Fc in 10 mM sodium acetate buffer at a pH of 5.5. The BSA (1 mg/mL) in the acetate buffer (pH 5.5) was then injected to reduce non-specific binding, followed by 1 M ethanolamine hydrochloride at a pH of 8.5 to quench residual NHS esters on the sensor chip surface. After that, binding rates were measured under a continuous flow at 20 μL/min. Immunoliposomes were injected at concentrations of 20 nM, 10 nM, 5 nM, 2.5 nM, 1.25 nM, and 0.625 nM. In between injections, the chip surface was regenerated with glycine hydrochloride (pH 3) until the response peak returned to the baseline. The binding activity and the kinetics were determined from a linear standard curve of binding slope versus concentration using the trace Drawer Software 9.2 [23,24,25].

### 2.7. Stability of CTX-OB-LPs

The stability of the conjugated liposomal formulations was assessed by measuring the particle size and zeta potential using DLS and drug content and by estimating entrapment efficiency using HPLC. Briefly, the formulation was stored at 4 °C and the samples were withdrawn weekly for 4 weeks, and the various stability parameters were evaluated over four weeks.

### 2.8. In Vitro Cell Studies

#### 2.8.1. Cytotoxicity Study

The in vitro cytotoxicity studies for the free drug (OB), unconjugated blank liposomes (blank LPs), CTX-conjugated liposomes (CTX-LPs), unconjugated OB liposomes (OB-LPs), and conjugated OB liposomes (CTX-OB-LPs) were performed using the MTT assay. For this assay, H1975 cells (5000 cells/well) were seeded in a 96-well plate and incubated (HeraCell 150i, Thermo Fisher, Waltham, MA, USA) at 37 °C, under 5% CO_2_, for 24 h. The cells were treated with varying concentrations of OB, blank LPs, CTX-LPs, OB-LPs, and CTX-OB-LPs, and DMSO was used as a positive control. The plates were treated for 72 h, followed by treatment removal and addition of 1 mg/mL MTT reagent solution. The plates were further incubated for 2 h at 37 °C, followed by the removal of the MTT solution and addition of DMSO to solubilize the formazan crystals. The absorbance was measured at 570 nm using a plate reader (Synergy H1, BioTek, Winooski, VT, USA). The percentage of viable cells was calculated as the absorbance ratio of the treatment wells to the untreated control wells. The half inhibitory concentration (IC_50_) was computed using the GraphPad Prism 8 software and a non-linear regression analysis. All the results are expressed as mean ± standard deviation (*n* = 3).

#### 2.8.2. Clonogenic Assay

The clonogenic assay was conducted to determine the ability of the cancer cells to survive and multiply from a single cell to form a colony. Briefly, H1975 cells (2000 cells/well) were seeded in a six-well plate and incubated for 24 h at 37 °C. The cells were treated with OB, OB-LPs, and CTX-OB-LPs (6.6 nM) for 72 h, and the untreated wells served as the control. The cells started forming colonies after an incubation period of 10 days. These colonies were fixed using 4% *w*/*v* paraformaldehyde for 30 min and stained for 2 h at room temperature with 0.1% *w*/*v* crystal violet solution. After staining, the wells were washed with sterile water and left to air-dry, and the images of the colonies were captured using a digital camera. The stained colonies were counted using a colony counting software, OpenCFU v3.9.0. The data were further analyzed using a one-way analysis of variance (ANOVA) and Tukey’s post hoc multiple comparison tests in GraphPad Prism 8. All results were expressed as mean ± standard deviation (*n* = 3), with levels of statistical significance considered at ** *p* < 0.001 and *** *p* < 0.0001.

#### 2.8.3. Scratch Assay

The scratch assay was performed to examine the cancer cells’ metastatic properties and migration capabilities. Briefly, H1975 cells (2 × 10^5^ cells/well) were seeded in a 24-well plate and incubated at 37 °C until they formed a confluent monolayer. Using a sterile 1000 µL micropipette tip, a uniform scratch was made along the diameter of the wells. The scratch images were captured before the treatments using an inverted microscope (Leica Microsystems Inc., Deerfield, IL, USA). Further, the cells were treated with OB, OB-LPs, and CTX-OB-LPs (6.6 nM) for 48 h, and the untreated wells served as the control. The wells were imaged at various time points, i.e., 12 h, 24 h, and 48 h, using a 5X objective on the inverted microscope. The widths of the created scratches were measured using the ImageJ Software 1.54g, and the data were analyzed using ANOVA and Tukey’s post hoc multiple comparison tests in GraphPad Prism 8, with all results expressed as mean ± standard deviation (*n* = 3). The levels of statistical significance were considered at * *p* < 0.05, ** *p* < 0.01, and **** *p* < 0.0001.

## 3. Results and Discussion

### 3.1. Physicochemical Characterization of OB-LPs and CTX-OB-LPs

The average particle size of the unconjugated OB liposomes (OB-LPs) was 130.38 ± 4.40 nm, while that of the conjugated OB liposomes (CTX-OB-LPs) was 153.97 ± 7.86 nm (Table 1). The slight increase in the particle size after conjugation can be attributed to the attachment of the antibody to the liposomal surface. Similar results were observed by Zheng et al., wherein the conjugation of the targeting ligand caused a slight increase in the size of the liposomal vesicles from 173 ± 13 nm to 186 ± 16 nm [26]. The particle size characteristics of the immunoliposomes (typically between 100 nm to 200 nm) can aid the evasion of lung clearance mechanisms, thereby prolonging the residence time at the tumor site, as reported in the literature [27,28]. The PDI of OB-LPs and CTX-OB-LPs was found to be 0.22 and 0.35, respectively, indicating a narrow and unimodal particle size distribution. The zeta potential value for the unconjugated liposomes was +4.39 ± 0.37 mV, which changed to −2.69 ± 0.98 mV for the conjugated liposomes. This could be attributed to the antibody conjugation onto the liposomal surface. The liposomal formulations (OB-LPs and CTX-OB-LPs) exhibited an encapsulation efficiency of 37.65 ± 2.48% and a drug loading of 33.05 μg (drug/mg of lipid). The antibody conjugation efficiency for the CTX-OB-LPs, as determined by BCA assay, was estimated at 87.53 ± 2.84%. Independent studies conducted by Petrilli et al. and Hamamichi et al. have reported similar findings for the liposomal antibody conjugation percentages using the BCA assay. The study performed by Petrilli et al. demonstrates that 94.5% of the cetuximab conjugated to the surface of the liposomes for the 5-fluouracil liposomal formulations [29]. Similarly, Hamamichi et al. report that the antibody conjugation percentages for different immunoliposomal formulations ranged from 60% to 76% [30].

### 3.2. Solid State Characterization

**Figure 2:**
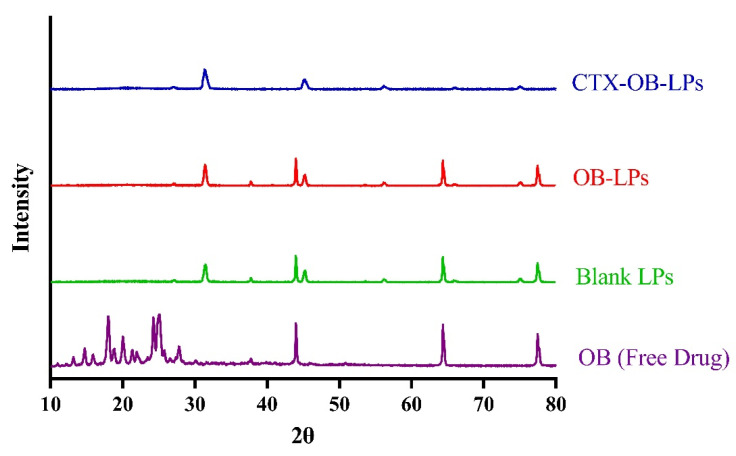
The XRD diffractogram of OB, blank formulation (blank LPs), unconjugated OB liposomes (OB-LPs), and conjugated OB liposomes (CTX-OB-LPs). The crystalline peaks of OB are absent in the XRD diffractogram of OB-LPs and CTX-OB-LPs, indicating drug encapsulation within the liposomes.

To further investigate the encapsulation of OB within liposomes, PXRD studies were performed. The crystalline nature of the pure drug, OB, was revealed from the XRD diffractogram, with intense peaks at 2θ values of 24.24°, 25.02°, 25.74°, and 27.76° (Figure 2). The disappearance of these intense peaks in the diffractograms for the unconjugated and conjugated OB liposomes suggests drug encapsulation within the liposomes.

### 3.3. In Vitro Drug Release

**Figure 3:**
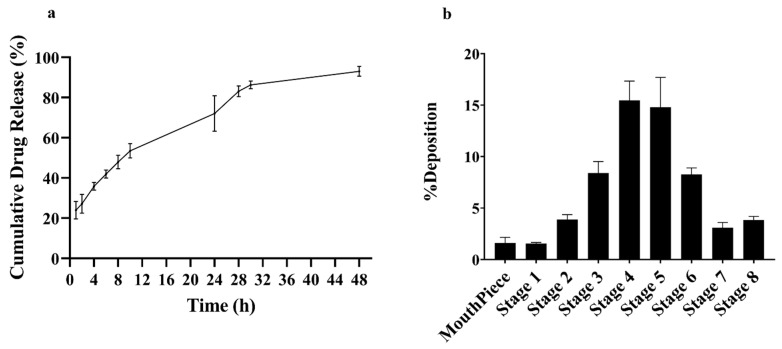
(**a**) Cumulative release profile for OB from immunoliposomes (CTX-OB-LPs) in phosphate buffer saline (PBS), pH 7.4. Data represents mean ± SD (*n* = 4). (**b**) In vitro aerosol deposition profile represented as percentage of drug deposited on each stage of next generation impactor (NGI). Data represents mean ± SD (*n* = 3).

The release of the drug (OB) from the conjugated liposomes (CTX-OB-LPs) was conducted at a pH of 7.4. It can be observed from Figure 3a that the liposomal formulation displayed a 24% initial burst release within an hour, followed by an almost 80% drug release from the liposomes within a period of 24 h and a complete release was observed within 48 h. A similar study was previously conducted by our group, wherein osimertinib liposomes were formulated via active and passive loading [22] and the studies reported that drug encapsulation within the lipid bilayer provides a quick release of the drug by diffusion [22].

### 3.4. Aerosolization of CTX-OB-LPs

Determining the aerodynamic characteristics of a formulation is essential to understand the fate of liposomal deposition in the respiratory airways. At a flow rate of 15 L/min, the deposition of the majority of the particles between stages 3 and 7 of the NGI was found to correlate with the deposition of the formulation in the lower respiratory tract. The percentage of the drug deposited at each stage is presented in Figure 3b. The mass median aerodynamic diameter (MMAD) of the OB immunoliposomes was found to be 3.22 μm (Table 2), which is well within the acceptable range (1–5 μm) for the deposition of aerosol droplets in the respiratory airways. The emitted dose (ED) for the CTX-OB-LPs was found to be 87.35 µg, while the fine particle fraction (FPF) was found to be greater than 80% *w*/*w*, suggesting deposition of the liposomal formulation into the respiratory airways of the lungs (Table 2). The GSD was 2.12, suggesting the generation of a polydisperse system for aerosol deposition [31].

#### Integrity of CTX-OB-LPs Post-Nebulization

In addition, we examined the influence of nebulization on the stability of the liposomes by evaluating the physicochemical characteristics of the CTX-OB-LP formulation after nebulization. We observed a 1.1-fold increase in the particle size of the liposomal formulation after nebulization, but there was no change observed in the polydispersity index of the formulation pre- and post-nebulization. Additionally, the zeta potential of the nanoparticulate systems slightly decreased from −3.93 ± 0.53 mV to −10.33 ± 1.52 mV for pre- and post-nebulization, respectively, possibly due to particle aggregation during aerosolization. Similar observations have been reported by Patil et al., wherein the authors observed nanoparticle aggregation which was attributed to particle contact during nebulization when utilizing jet nebulizers [32].

### 3.5. Binding Kinetics of EGFR with CTX and CTX-OB-LPs

**Figure 4:**
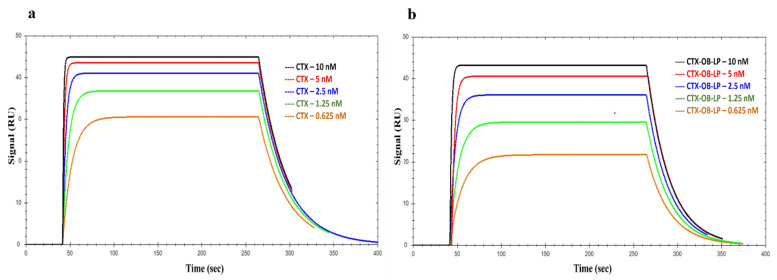
Kinetic analysis using SPR for the (**a**) binding of antibodies (CTX) to the EGFR protein and (**b**) binding of CTX-OB-LPs to the EGFR protein. Data were fitted using the TraceDrawer software at various concentrations injected at 20 μL/min over EGFR immobilized on the sensor’s surface. Data represents mean ± SD (*n* = 3).

Cetuximab was used in increasing concentrations, running from low to high order, using kinetic analysis. The PBS buffer was used to make serial dilutions, and the concentrations injected were 20 nM, 10 nM, 5 nM, 2.5 nM, 1.25 nM, and 0.625 nM. A 0 nM sample (blank buffer) was also injected for reference subtraction. The data were analyzed with varying concentrations of cetuximab. The representative sensorgrams of the evaluated data for the antibody are presented in Figure 4a. The sensorgrams were fitted using a 1:1 binding model. The curves showed a slow dissociation, with an apparent k_a_ (association constant) of 2.13 × 10^8^ M^−1^ s^−1^ and a k_d_ (dissociation constant) of 4.75 × 10^−2^ s^−1^, resulting in a calculated K_D_ (equilibrium dissociation constant, K_D_ = k_d_/k_a_) of 0.22 nM, which is in good agreement with the published literature [33,34,35]. The equilibrium binding constants for cetuximab and sEGFR interactions are reported in Table 3 along with literature values for comparison.

Similarly, cetuximab-conjugated liposomes were diluted in an increasing concentration of 0.1 K_D_ to 10 K_D_ of CTX using PBS buffer and injected. The representative sensorgrams of the evaluated data for the immunoliposomes EGFR binding are presented in Figure 4b. The sensorgrams were fitted using a 1:1 binding model. The curves showed a slow dissociation, with an apparent k_a_ of 5.38 × 10^8^ M^−1^ s^−1^ and a k_d_ of 6.02 × 10^−2^ s^−1^, resulting in a calculated K_D_ of 0.12 nM which is in good agreement with previously reported K_D_ values for the unconjugated CTX antibody [33,34,35]. Thus, from the obtained results, it can be concluded that, upon conjugation of the antibody to the liposomes, the CTX binding affinity to EGFR is retained and allows for a targeted delivery of the liposomal formulation to the EGFR receptor.

### 3.6. In Vitro Cell Culture Studies

#### 3.6.1. Cytotoxicity Study

**Figure 5:**
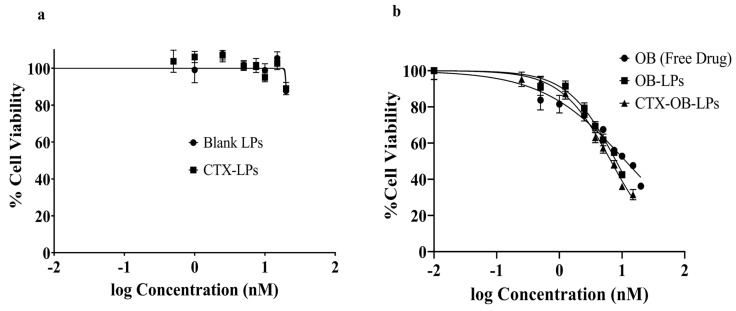
Cytotoxicity studies after 72 h treatment, as determined using the MTT assay in the H1975 cell line. (**a**) Blank liposomes (blank LPs) and CTX-conjugated liposomes (CTX-LPs); (**b**) OB, unconjugated OB liposomes (OB-LPs), and conjugated OB liposomes (CTX-OB-LPs). Data represents mean ± SD (*n* = 3).

The cytotoxicity of the formulations was studied in the NSCLC cell line, H1975 bearing the EGFR mutations, T790M, and L858R (Figure 5). The free drug, OB, showed an IC_50_ value of 11.24 ± 0.49 nM, thereby indicating the potency of the drug effect on this cell line. The conjugated OB liposomes (CTX-OB-LPs) resulted in a 1.7-fold reduction in the IC_50_ value (IC_50_ of 6.73 ± 0.78 nM) compared to the free drug (*p* < 0.005). Further, there was a 1.2-fold reduction in the IC_50_ value for the conjugated OB liposomes compared to the unconjugated OB liposomes (OB-LPs) (IC_50_ of 8.07 ± 0.46 nM, *p* < 0.05). The increased cytotoxicity of the immunoliposomes could be due to the antibody attached to the liposomal surface that serves the dual role of acting as a targeting ligand as well as providing synergistic activity. This 1.7-fold increase in potency highlights the ability of the antibody-targeted liposomal formulations to improve cytotoxicity profiles. Similar findings have been reported by Canato et al. and Zalba et al., wherein the targeted liposomal formulations displayed a higher cytotoxic activity compared to the non-targeted formulations. The study performed by Canato’s group demonstrated a 1.4-fold reduction in the IC_50_ value for the targeted formulation compared to the non-targeted formulation in the HER2- cell line [36]. Also, the study by Zalba et al. reveals that the cetuximab-targeted liposomes exhibited a significantly lower IC_50_ value of 16.64 μM compared to 28.67 μM for the free drug when evaluated in the HCT-116 cell line [37]. In addition, the blank LPs and CTX-LPs showed > 90% cell viability at all concentrations tested, confirming that the cytotoxicity observed was due to the drug being entrapped in LPs and was not due to LPs themselves.

#### 3.6.2. Clonogenic Assay

**Figure 6:**
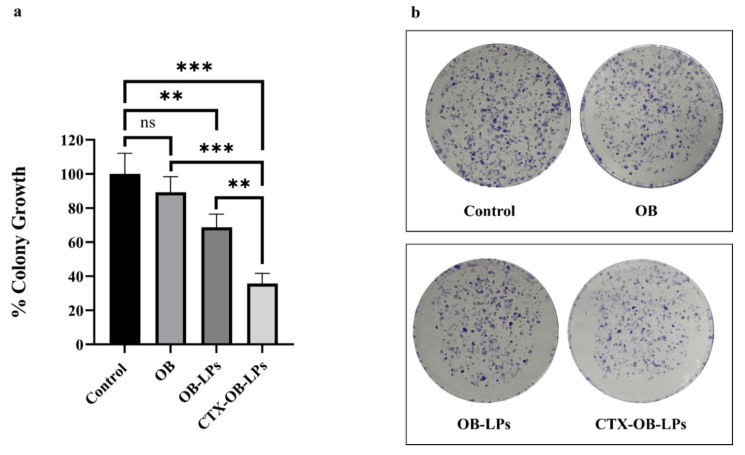
Colony-forming ability of H1975 cells under treatment for 72 h, followed by a 10-day incubation in fresh media. (**a**) Quantitative analysis of the clonogenic nature of the H1975 cells after treatment with OB, unconjugated OB liposomes (OB-LPs), and conjugated OB liposomes (CTX-OB-LPs). (**b**) Images of the colonies after crystal violet staining. The data are expressed as % colony growth versus the respective treatment. Data represents mean ± SD (*n* = 3). *** *p* < 0.0001 and ** *p* < 0.001; ns—non-significant.

The clonogenic assay served as an indicator to understand the effect of unconjugated and conjugated OB liposomes on the clonal expansion and metastatic potential of the H1975 cells. There was no significant difference observed between the free drug (OB) and the control (Figure 6). Furthermore, treatment with unconjugated (OB-LPs) and conjugated liposomes (CTX-OB-LPs) showed a significant reduction in the cell clonal expansion compared to the control. It can be observed in Figure 6a that approximately 90% of the colonies survived the treatment with the free drug (OB) compared to the control. However, a significant reduction in colony growth of ~65% and 35% was observed in unconjugated (OB-LPs) and conjugated liposomes (CTX-OB-LPs), respectively, when compared to the control (*p* < 0.001 and *p* < 0.0001). Furthermore, a significant (*p* < 0.001) colony growth reduction of ~20% was observed in the conjugated liposomes (CTX-OB-LPs) relative to the unconjugated liposomes (OB-LPs). These results are comparable to the findings by Yang et al., where they investigated the effect of SATB1 siRNA encapsulated immunoliposomes on gastric cancer-initiating cells. They reported that the antibody-conjugated liposomal formulations reduced the growth of the colonies by ~27% compared to the unconjugated liposomes in gastric cancer cell lines [38].

#### 3.6.3. Scratch Assay

**Figure 7:**
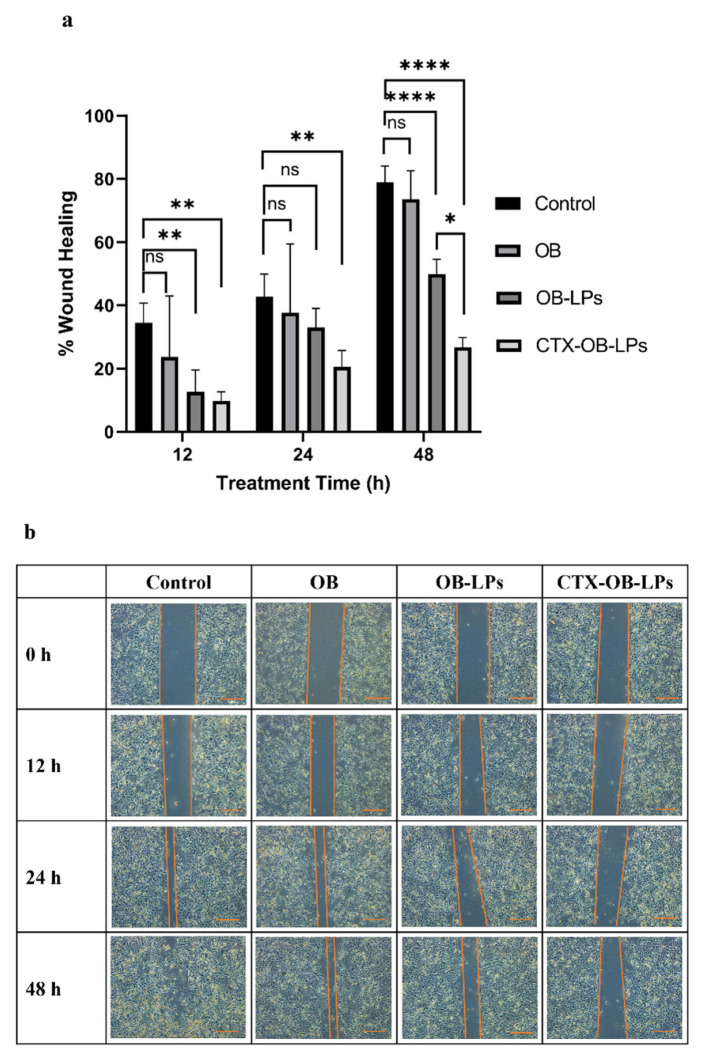
(**a**) Scratch assay analysis of the H1975 cell line, shown as % of wound healing over time after treatment with OB, unconjugated OB liposomes (OB-LPs), and conjugated OB liposomes (CTX-OB-LPs). Data represents mean ± SD (*n* = 3). **** *p* < 0.0001, ** *p* < 0.01, and * *p* < 0.05; ns—non-significant. (**b**) Effect of OB and liposomal formulations (OB-LPs and CTX-OB-LPs) on the metastatic potential of the H1975 cell line. Representative microscopic images of the scratch after the following treatment times are provided: 0 h, 12 h, 24 h, and 48 h. Scale bar 400 µm.

The scratch assay was conducted to determine the effect of the free drug (OB), unconjugated liposomes (OB-LPs), and conjugated liposomes (CTX-OB-LPs) on the migratory potential of the H1975 cells. It can be observed from Figure 7a, b that after the 48 h incubation, the scratch closure was significantly reduced for the unconjugated (OB-LPs) and conjugated liposomes (CTX-OB-LPs) when compared to the control group, indicating the potential for inhibition of cell migration. It was observed that the wound closure for the control group was 81.9 ± 7.0%. Furthermore, the control and the free drug groups displayed an almost complete scratch closure when compared to the unconjugated and conjugated liposomes. This could be attributed to the controlled and prolonged release of the drug from the liposomes over 48 h. Additionally, after the 48 h incubation time, there was a significant reduction of 26.75 ± 3.06% in the wound closure of the conjugated liposomes (CTX-OB-LPs) which was lower than that found for the unconjugated liposomes (OB-LPs), i.e., 49.89 ± 4.65%, thereby demonstrating a greater inhibitory effect. Wu et al. investigated the effect of folate-modified liposomes on cisplatin-resistant lung cancer cells, and their data show similar findings for the scratch assay, with the folate-targeted liposomal formulation showing minimal migration compared to the non-targeted formulations [39].

### 3.7. Stability of CTX-OB-LPs

**Figure 8:**
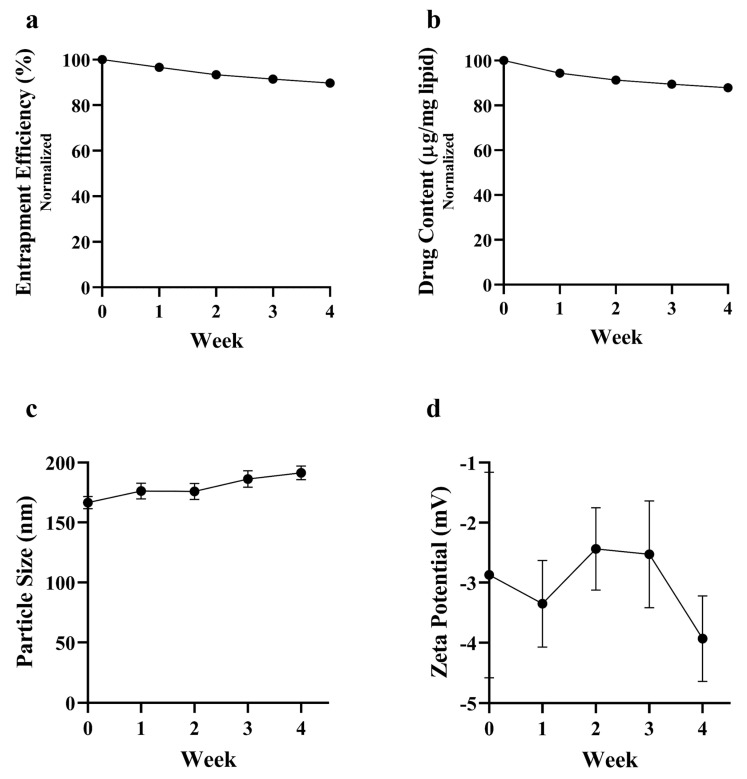
Stability data for CTX-OB-LPs when stored at 4 °C. (**a**) % entrapment efficiency (EE); (**b**) drug content; (**c**) particle size; (**d**) zeta potential.

The stability of the liposomal formulation is essential to determine the shelf life of the product. Various stability parameters including particle size, zeta potential, % EE, and drug content were assessed weekly for samples stored at 4 °C. There was not much reduction observed in % EE (<10%) and drug content (12%) over the period of four weeks (Figure 8a,b). The insignificant loss of drug from the liposomes could be attributed to the minimal leakage of the drug from the liposomal structure. There was a slight increase in the particle size over weeks 3 and 4 (Figure 8c) which could be identified as aggregation of the liposomal particles during storage. The zeta potential for the formulation essentially remained the same with no major fluctuations (Figure 8d). To address the issue of particle size and prolong the stability of the liposomes, the formulation could either be spray-dried or lyophilized.

## 4. Conclusions

The development of OB immunoliposomes to enhance drug delivery to the tumor site and serve as a solution to OB resistance is a promising approach. In this research study, antibody-conjugated OB liposomes are successfully developed and exhibit a particle size of ~150 nm while achieving a high antibody conjugation efficiency (87%). These immunoliposomes exhibit efficient drug release, with a greater than 80% drug release from the liposomes within 24 h, and excellent aerosolization properties, with an FPF of ~90%, suggesting deposition in respiratory airways. Furthermore, in vitro studies of the immunoliposomes show enhanced cytotoxicity (~1.2-fold reduction in IC_50_ values for the immunoliposomes compared to the unconjugated liposomes) and significantly reduced tumor cell migration compared to the free drug and the unconjugated liposomes. Overall, EGFR-targeting inhalable immunoliposomes are successfully developed and could contribute to greater anti-tumor efficacy for NSCLC treatment.

## Figures and Tables

**Table 1:** Physicochemical characterization of osimertinib unconjugated (OB-LPs) and conjugated immunoliposomes (CTX-OB-LPs). Data represents mean ± SD (*n* = 5).

Formulation	Size (nm)	PDI (a.u)	Zeta Potential (mV)
OB-LPs (pre-conjugation)	130.38 ± 4.40	0.22 ± 0.01	+4.39 ± 0.37
CTX-OB-LPs (post-conjugation)	153.97 ± 7.86	0.35 ± 0.01	−2.69 ± 0.98

**Table 2:** In vitro aerosolization properties for osimertinib immunoliposomes (CTX-OB-LPs). Data represents mean ± SD (*n* = 3).

Aerodynamic Properties	OB Immunoliposomes (CTX-OB-LPs)
MMAD (μm)	3.22 ± 0.12
FPF (%)	88.43 ± 0.38
GSD	2.12 ± 0.08
ED (μg)	87.35 ± 8.87

**Table 3:** Summary of equilibrium binding constants for the cetuximab/sEGFR interaction.

Our studies (Nicoya Rev4)—cetuximab *	0.22 ± 0.0002 nM
Our studies (Nicoya Rev4)—immunoliposomes *	0.12 ± 0.0005 nM
Kankanala et al., 2009 (Biocore)—cetuximab [33]	0.15 ± 0.05 nM
Neil I. Goldstein et al., 1995 (Biacore)—cetuximab [35]	0.2 nM

* Data represents mean ± SD (*n* = 3).

## Data Availability

Any datasets generated during the current study are available from the corresponding author upon reasonable request.
